# Supplementary material for: Hot spot prediction in protein-protein interactions by an ensemble system
Source: BMC Syst Biol. 2018 Dec 31;12(Suppl 9):132. doi: 10.1186/s12918-018-0665-8 (PMC6311905; doi:10.1186/s12918-018-0665-8)
Supplement: Supplementary file 2 — All relevant MATLAB code. A simple Matlab implement of our predictor. (ZIP 19,742 kb) [file 12918_2018_665_MOESM2_ESM.zip › Additional file 2/Read ASA/ASA website/dbMPIKT/4BFIB.html]

 NetSurfP - Protein Surface Accessibility and Secondary Structure Predictions

  

|  |  |  |
| --- | --- | --- |
|  |  | NetSurfP - Protein Surface Accessibility and Secondary Structure Predictions  Technical University of Denmark |

  


---

```
# For publication of results, please cite:
# A generic method for assignment of reliability scores applied to solvent accessibility predictions.
# Bent Petersen, Thomas Nordahl Petersen, Pernille Andersen, Morten Nielsen and Claus Lundegaard
# BMC Structural Biology 2009, 9:51 doi:10.1186/1472-6807-9-51
#
# Column 1: Class assignment - B for buried or E for Exposed - Threshold: 25% exposure, but not based on RSA
# Column 2: Amino acid
# Column 3: Sequence name
# Column 4: Amino acid number
# Column 5: Relative Surface Accessibility - RSA
# Column 6: Absolute Surface Accessibility
# Column 7: Z-fit score for RSA prediction
# Column 8: Probability for Alpha-Helix
# Column 9: Probability for Beta-strand
# Column 10: Probability for Coil
E Q  4BFIB                  1    0.797 142.308   1.367   0.005   0.015   0.979
B V  4BFIB                  2    0.192  29.434  -2.282   0.003   0.718   0.279
E E  4BFIB                  3    0.644 112.437   0.740   0.002   0.816   0.182
E V  4BFIB                  4    0.486  74.744  -0.006   0.003   0.718   0.279
E V  4BFIB                  5    0.449  68.950   0.746   0.005   0.336   0.660
E T  4BFIB                  6    0.531  73.677   0.382   0.004   0.138   0.858
E Q  4BFIB                  7    0.619 110.643   0.967   0.004   0.197   0.799
E D  4BFIB                  8    0.597  86.085   0.555   0.004   0.420   0.576
B E  4BFIB                  9    0.147  25.716   0.785   0.002   0.816   0.182
E R  4BFIB                 10    0.498 114.111   1.120   0.001   0.959   0.040
B K  4BFIB                 11    0.075  15.530  -0.004   0.001   0.959   0.040
E A  4BFIB                 12    0.468  51.530   1.336   0.001   0.900   0.099
E L  4BFIB                 13    0.359  65.660   1.010   0.004   0.420   0.576
E H  4BFIB                 14    0.689 125.311  -0.588   0.005   0.045   0.951
E T  4BFIB                 15    0.555  77.020   1.164   0.005   0.045   0.951
E T  4BFIB                 16    0.569  78.962   1.316   0.004   0.514   0.481
B A  4BFIB                 17    0.043   4.739   0.439   0.001   0.900   0.099
E S  4BFIB                 18    0.379  44.384   1.683   0.001   0.959   0.040
B L  4BFIB                 19    0.046   8.423   1.043   0.001   0.959   0.040
E R  4BFIB                 20    0.352  80.631   1.476   0.001   0.900   0.099
B C  4BFIB                 21    0.020   2.808   0.661   0.001   0.900   0.099
E S  4BFIB                 22    0.421  49.400   1.229   0.001   0.900   0.099
B L  4BFIB                 23    0.081  14.904  -0.553   0.001   0.900   0.099
E K  4BFIB                 24    0.486  99.888   0.686   0.004   0.514   0.481
E T  4BFIB                 25    0.618  85.772  -1.918   0.004   0.085   0.910
E S  4BFIB                 26    0.604  70.765  -1.314   0.005   0.015   0.979
E Q  4BFIB                 27    0.603 107.767  -2.046   0.003   0.003   0.994
E E  4BFIB                 28    0.661 115.512  -0.075   0.005   0.045   0.951
E P  4BFIB                 29    0.299  42.471  -0.486   0.005   0.336   0.660
B L  4BFIB                 30    0.191  34.917   0.045   0.003   0.718   0.279
E I  4BFIB                 31    0.335  61.920   0.672   0.001   0.900   0.099
B V  4BFIB                 32    0.028   4.350   0.573   0.001   0.959   0.040
B T  4BFIB                 33    0.207  28.669   1.515   0.001   0.959   0.040
B W  4BFIB                 34    0.027   6.542   0.394   0.001   0.959   0.040
B Q  4BFIB                 35    0.205  36.577   1.423   0.001   0.959   0.040
B K  4BFIB                 36    0.174  35.730   0.655   0.001   0.959   0.040
E K  4BFIB                 37    0.412  84.646   0.133   0.004   0.514   0.481
E K  4BFIB                 38    0.606 124.716  -0.871   0.004   0.085   0.910
E A  4BFIB                 39    0.655  72.148  -1.731   0.005   0.015   0.979
E V  4BFIB                 40    0.704 108.205  -1.703   0.005   0.015   0.979
E S  4BFIB                 41    0.538  63.089  -0.863   0.004   0.085   0.910
E P  4BFIB                 42    0.343  48.629  -0.575   0.005   0.336   0.660
E E  4BFIB                 43    0.403  70.352  -0.692   0.004   0.616   0.381
B N  4BFIB                 44    0.265  38.796   0.231   0.002   0.816   0.182
B M  4BFIB                 45    0.103  20.550  -0.069   0.001   0.900   0.099
B V  4BFIB                 46    0.146  22.379  -0.343   0.001   0.900   0.099
B T  4BFIB                 47    0.184  25.465  -1.088   0.001   0.900   0.099
B Y  4BFIB                 48    0.183  39.022  -0.989   0.003   0.718   0.279
B S  4BFIB                 49    0.269  31.562  -0.822   0.021   0.451   0.528
E K  4BFIB                 50    0.482  99.065  -1.266   0.019   0.141   0.840
E T  4BFIB                 51    0.564  78.282  -1.731   0.019   0.141   0.840
E H  4BFIB                 52    0.473  86.093  -1.074   0.020   0.205   0.775
E G  4BFIB                 53    0.495  38.956  -1.054   0.021   0.279   0.699
E V  4BFIB                 54    0.467  71.701  -1.050   0.022   0.552   0.426
E V  4BFIB                 55    0.578  88.854  -0.493   0.021   0.756   0.223
E I  4BFIB                 56    0.333  61.549  -0.503   0.023   0.655   0.322
E Q  4BFIB                 57    0.396  70.654  -0.609   0.022   0.359   0.619
E P  4BFIB                 58    0.534  75.775  -1.640   0.019   0.141   0.840
E A  4BFIB                 59    0.627  69.051  -0.282   0.019   0.141   0.840
B Y  4BFIB                 60    0.230  49.151  -0.889   0.004   0.197   0.799
E K  4BFIB                 61    0.541 111.201  -0.193   0.005   0.262   0.733
E D  4BFIB                 62    0.329  47.394  -1.392   0.004   0.420   0.576
B R  4BFIB                 63    0.178  40.854  -0.383   0.003   0.718   0.279
B I  4BFIB                 64    0.080  14.800  -1.071   0.001   0.900   0.099
E N  4BFIB                 65    0.272  39.821  -0.165   0.001   0.959   0.040
B V  4BFIB                 66    0.101  15.462  -0.693   0.001   0.959   0.040
E T  4BFIB                 67    0.256  35.577  -0.201   0.001   0.900   0.099
E E  4BFIB                 68    0.456  79.751  -1.227   0.003   0.718   0.279
E L  4BFIB                 69    0.481  87.980  -1.108   0.004   0.420   0.576
E G  4BFIB                 70    0.608  47.873  -1.800   0.004   0.085   0.910
E L  4BFIB                 71    0.492  90.085  -1.524   0.004   0.085   0.910
E W  4BFIB                 72    0.561 134.872  -1.223   0.004   0.197   0.799
E N  4BFIB                 73    0.435  63.640   0.090   0.004   0.420   0.576
B S  4BFIB                 74    0.079   9.306  -0.639   0.003   0.718   0.279
B S  4BFIB                 75    0.162  18.998   0.094   0.001   0.900   0.099
B I  4BFIB                 76    0.023   4.218   0.845   0.001   0.959   0.040
E T  4BFIB                 77    0.241  33.482   1.679   0.001   0.959   0.040
B F  4BFIB                 78    0.033   6.663  -0.124   0.001   0.959   0.040
E W  4BFIB                 79    0.440 105.772   1.518   0.002   0.816   0.182
E N  4BFIB                 80    0.535  78.251   0.661   0.004   0.616   0.381
B T  4BFIB                 81    0.100  13.939   0.623   0.004   0.514   0.481
E T  4BFIB                 82    0.470  65.161   1.145   0.004   0.514   0.481
E L  4BFIB                 83    0.482  88.254   0.301   0.004   0.420   0.576
E E  4BFIB                 84    0.583 101.798   0.167   0.005   0.262   0.733
E D  4BFIB                 85    0.290  41.803  -0.146   0.004   0.138   0.858
E E  4BFIB                 86    0.605 105.728   0.004   0.004   0.138   0.858
B G  4BFIB                 87    0.191  15.047  -1.097   0.003   0.718   0.279
E C  4BFIB                 88    0.403  56.609   2.166   0.001   0.959   0.040
B Y  4BFIB                 89    0.039   8.334   0.381   0.001   0.959   0.040
B M  4BFIB                 90    0.240  48.004   2.165   0.001   0.959   0.040
B C  4BFIB                 91    0.013   1.825   1.612   0.001   0.959   0.040
B L  4BFIB                 92    0.195  35.741   1.724   0.001   0.959   0.040
B F  4BFIB                 93    0.035   7.045  -0.447   0.001   0.959   0.040
E N  4BFIB                 94    0.319  46.658   0.905   0.001   0.959   0.040
B T  4BFIB                 95    0.177  24.494  -0.606   0.001   0.900   0.099
E F  4BFIB                 96    0.430  86.221  -0.056   0.004   0.197   0.799
E G  4BFIB                 97    0.698  54.956  -1.208   0.005   0.045   0.951
E S  4BFIB                 98    0.628  73.566  -1.219   0.005   0.045   0.951
E Q  4BFIB                 99    0.418  74.726  -1.100   0.005   0.262   0.733
E K  4BFIB                100    0.511 105.092  -0.266   0.003   0.718   0.279
E V  4BFIB                101    0.328  50.414   0.269   0.001   0.900   0.099
E S  4BFIB                102    0.392  45.931  -0.321   0.001   0.900   0.099
B G  4BFIB                103    0.182  14.347  -0.118   0.001   0.959   0.040
E T  4BFIB                104    0.292  40.528   0.565   0.001   0.959   0.040
B A  4BFIB                105    0.054   6.006   0.547   0.001   0.959   0.040
E C  4BFIB                106    0.284  39.874   1.519   0.001   0.959   0.040
B L  4BFIB                107    0.035   6.482  -0.296   0.001   0.959   0.040
E T  4BFIB                108    0.384  53.289   0.867   0.001   0.900   0.099
E L  4BFIB                109    0.322  59.050  -0.109   0.002   0.816   0.182
E Y  4BFIB                110    0.458  97.768  -0.269   0.005   0.336   0.660
E V  4BFIB                111    0.385  59.190  -0.822   0.004   0.197   0.799
E Q  4BFIB                112    0.501  89.461  -0.146   0.004   0.085   0.910
B P  4BFIB                113    0.201  28.522  -0.969   0.004   0.197   0.799
E I  4BFIB                114    0.564 104.359   0.122   0.004   0.616   0.381
B V  4BFIB                115    0.140  21.533  -0.127   0.002   0.816   0.182
E H  4BFIB                116    0.461  83.892   0.126   0.002   0.816   0.182
E L  4BFIB                117    0.301  55.040  -0.972   0.002   0.816   0.182
E H  4BFIB                118    0.494  89.822  -0.444   0.004   0.616   0.381
E Y  4BFIB                119    0.368  78.684  -1.433   0.005   0.336   0.660
E N  4BFIB                120    0.543  79.481  -0.614   0.004   0.197   0.799
E Y  4BFIB                121    0.490 104.798  -1.115   0.004   0.138   0.858
E F  4BFIB                122    0.636 127.705  -1.548   0.005   0.045   0.951
E E  4BFIB                123    0.729 127.409  -1.491   0.005   0.045   0.951
E D  4BFIB                124    0.525  75.638  -0.571   0.004   0.197   0.799
E H  4BFIB                125    0.466  84.784   0.590   0.002   0.816   0.182
B L  4BFIB                126    0.092  16.809   0.139   0.001   0.959   0.040
E N  4BFIB                127    0.329  48.107   1.224   0.001   0.959   0.040
B I  4BFIB                128    0.053   9.768   0.585   0.001   0.959   0.040
B T  4BFIB                129    0.247  34.273   1.173   0.001   0.959   0.040
B C  4BFIB                130    0.033   4.703  -0.007   0.001   0.959   0.040
E S  4BFIB                131    0.402  47.091   0.806   0.001   0.959   0.040
B A  4BFIB                132    0.093  10.238  -1.549   0.002   0.816   0.182
E T  4BFIB                133    0.627  86.965  -0.081   0.005   0.262   0.733
E A  4BFIB                134    0.532  58.615  -2.045   0.004   0.085   0.910
E R  4BFIB                135    0.479 109.737  -0.027   0.004   0.197   0.799
B P  4BFIB                136    0.213  30.211  -0.605   0.004   0.138   0.858
E A  4BFIB                137    0.515  56.709   0.810   0.004   0.138   0.858
B P  4BFIB                138    0.121  17.142  -0.503   0.004   0.420   0.576
E A  4BFIB                139    0.419  46.174   1.004   0.002   0.816   0.182
B I  4BFIB                140    0.056  10.341   0.768   0.001   0.959   0.040
E S  4BFIB                141    0.352  41.278   1.448   0.001   0.959   0.040
B W  4BFIB                142    0.036   8.706   0.036   0.001   0.959   0.040
E K  4BFIB                143    0.367  75.471   1.431   0.001   0.900   0.099
E G  4BFIB                144    0.394  30.976   0.815   0.004   0.514   0.481
E T  4BFIB                145    0.683  94.774  -0.310   0.018   0.019   0.964
E G  4BFIB                146    0.718  56.491  -1.616   0.018   0.019   0.964
E T  4BFIB                147    0.624  86.563   0.024   0.004   0.420   0.576
E G  4BFIB                148    0.544  42.797  -0.127   0.004   0.616   0.381
B I  4BFIB                149    0.165  30.451  -0.616   0.004   0.420   0.576
E E  4BFIB                150    0.515  89.988  -0.204   0.005   0.262   0.733
E N  4BFIB                151    0.531  77.709  -1.463   0.004   0.085   0.910
E S  4BFIB                152    0.528  61.882  -1.553   0.004   0.085   0.910
E T  4BFIB                153    0.550  76.271  -1.045   0.005   0.336   0.660
E E  4BFIB                154    0.522  91.106  -0.100   0.002   0.816   0.182
E S  4BFIB                155    0.464  54.439  -0.341   0.001   0.900   0.099
E H  4BFIB                156    0.477  86.803  -0.171   0.001   0.900   0.099
E F  4BFIB                157    0.432  86.642  -0.211   0.002   0.816   0.182
E H  4BFIB                158    0.430  78.290  -0.243   0.004   0.514   0.481
E S  4BFIB                159    0.674  78.946  -1.474   0.004   0.085   0.910
E N  4BFIB                160    0.769 112.640  -1.006   0.005   0.045   0.951
E G  4BFIB                161    0.395  31.063  -1.101   0.004   0.197   0.799
E T  4BFIB                162    0.404  55.979  -0.090   0.003   0.718   0.279
B T  4BFIB                163    0.185  25.618  -0.404   0.001   0.900   0.099
E S  4BFIB                164    0.295  34.633  -0.110   0.001   0.900   0.099
B V  4BFIB                165    0.153  23.455  -0.362   0.001   0.959   0.040
E T  4BFIB                166    0.329  45.591  -0.264   0.001   0.959   0.040
B S  4BFIB                167    0.079   9.282  -1.121   0.001   0.900   0.099
B I  4BFIB                168    0.228  42.088   0.403   0.001   0.959   0.040
B L  4BFIB                169    0.032   5.878   0.321   0.001   0.959   0.040
E R  4BFIB                170    0.265  60.799   1.513   0.001   0.959   0.040
B V  4BFIB                171    0.045   6.932  -0.169   0.001   0.959   0.040
E K  4BFIB                172    0.377  77.590   1.153   0.001   0.900   0.099
E D  4BFIB                173    0.433  62.410  -0.472   0.002   0.816   0.182
E P  4BFIB                174    0.278  39.448   0.014   0.004   0.420   0.576
E K  4BFIB                175    0.535 110.070   0.137   0.005   0.336   0.660
E T  4BFIB                176    0.551  76.382  -0.016   0.005   0.262   0.733
E Q  4BFIB                177    0.601 107.392  -0.762   0.004   0.085   0.910
E V  4BFIB                178    0.494  76.005  -1.009   0.005   0.045   0.951
E G  4BFIB                179    0.614  48.298  -0.798   0.004   0.085   0.910
B K  4BFIB                180    0.251  51.672  -0.462   0.004   0.616   0.381
E E  4BFIB                181    0.387  67.574   1.961   0.001   0.900   0.099
B V  4BFIB                182    0.043   6.640   0.693   0.001   0.959   0.040
B I  4BFIB                183    0.203  37.573   1.604   0.001   0.959   0.040
B C  4BFIB                184    0.013   1.825   1.547   0.001   0.959   0.040
B Q  4BFIB                185    0.211  37.756   1.809   0.001   0.959   0.040
B V  4BFIB                186    0.025   3.781   0.512   0.001   0.959   0.040
E L  4BFIB                187    0.360  65.989   1.070   0.001   0.959   0.040
B Y  4BFIB                188    0.133  28.465   0.185   0.004   0.616   0.381
E L  4BFIB                189    0.577 105.704   0.508   0.005   0.045   0.951
E G  4BFIB                190    0.578  45.481  -1.226   0.005   0.045   0.951
E N  4BFIB                191    0.597  87.430  -0.916   0.005   0.262   0.733
E V  4BFIB                192    0.561  86.210  -0.421   0.004   0.616   0.381
E I  4BFIB                193    0.412  76.146   0.137   0.002   0.816   0.182
E D  4BFIB                194    0.567  81.762   0.136   0.001   0.900   0.099
B Y  4BFIB                195    0.217  46.437   1.079   0.001   0.900   0.099
E K  4BFIB                196    0.495 101.842   0.906   0.001   0.959   0.040
B Q  4BFIB                197    0.064  11.395   0.401   0.001   0.959   0.040
E S  4BFIB                198    0.422  49.435   1.710   0.001   0.959   0.040
B L  4BFIB                199    0.058  10.601   0.453   0.001   0.900   0.099
E D  4BFIB                200    0.424  61.055   0.703   0.002   0.816   0.182
B K  4BFIB                201    0.215  44.123   0.354   0.022   0.552   0.426
E G  4BFIB                202    0.607  47.802  -0.699   0.019   0.141   0.840
E S  4BFIB                203    0.451  52.810  -1.482   0.018   0.047   0.935
E T  4BFIB                204    0.531  73.664  -1.015   0.018   0.047   0.935
E R  4BFIB                205    0.530 121.439  -1.556   0.018   0.047   0.935
E H  4BFIB                206    0.523  95.152  -1.079   0.018   0.088   0.893
E H  4BFIB                207    0.379  68.922  -0.896   0.020   0.205   0.775
E H  4BFIB                208    0.502  91.296  -0.355   0.021   0.279   0.699
E H  4BFIB                209    0.557 101.300  -1.021   0.022   0.359   0.619
E H  4BFIB                210    0.421  76.634   0.015   0.020   0.205   0.775
E H  4BFIB                211    0.749 136.261  -1.538   0.003   0.003   0.994

Explain the output.  Go back.
```

---
